# Analyzing the Bills-Voting Dynamics and Predicting Corruption-Convictions Among Brazilian Congressmen Through Temporal Networks

**DOI:** 10.1038/s41598-019-53252-9

**Published:** 2019-11-14

**Authors:** Tiago Colliri, Liang Zhao

**Affiliations:** 1 Dept. of Computer Science, ICMC-USP, Sao Carlos, Brazil; 2 Dept. of Computing and Mathematics, FFCLRP-USP, Ribeirao Preto, Brazil

**Keywords:** Computer science, Computational science

## Abstract

In this paper, we propose a network-based technique to analyze bills-voting data comprising the votes of Brazilian congressmen for a period of 28 years. The voting sessions are initially mapped into static networks, where each node represents a congressman and each edge stands for the similarity of votes between a pair of congressmen. Afterwards, the constructed static networks are converted to temporal networks. Our analyses on the temporal networks capture some of the main political changes happened in Brazil during the period of time under consideration. Moreover, we find out that the bills-voting networks can be used to identify convicted politicians, who commit corruption or other financial crimes. Therefore, we propose two conviction prediction methods, one is based on the highest weighted convicted neighbor and the other is based on link prediction techniques. It is a surprise to us that the high accuracy (up to 90% by the link prediction method) on predicting convictions is achieved only through bills-voting data, without taking into account any financial information beforehand. Such a feature makes possible to monitor congressmen just by considering their legal public activities. In this way, our work contributes to the large scale public data study using complex networks.

## Introduction

Complex networks refer to large scale *graphs* with non-trivial connection patterns^1^. Some examples include the internet^2^, biological neural networks^3^, social networks^4^, food chains^5^, blood distribution networks^6^ and power grid distribution networks^7^. Complex networks have also been applied to data modeling and simulations, such as the spreading of infectious diseases^8^ and social contagion^9^, and failures and attacks in the WWW and the internet^10^. Besides, there are currently several network-based models that have been designed to perform machine learning tasks, such as clustering^11^, classification^12^ and regression^13^. More recently, *temporal networks* have been introduced, which allows us to take into account the *time* dimension as well in the study of graphs. Examples of real-world systems, which can be modeled through temporal networks, include social networks, one-to-many information dissemination (such as in emails or blogs, for instance), cell-biology networks, brain networks, traffic networks, and mobile communication networks^14^.

In the last years, governments around the world have been trying to increase their transparency by making large amount of public administration data available to the population. This phenomenon had triggered the development of new methods specifically designed for the analysis of such kind of data. Within this context, network-based techniques have also been applied to politics-related data, such as the analysis of the legislators’ relations through bill co-sponsorship data^15,16^ and through roll-call voting data^17–20^. A comprehensive review on this topic has been made by Victor *et al*.^21^. Besides, there are also applications on the analysis of networks for crimes-related purposes. Wachs *et al*.^22^ studied the social aspects of corruption by relating the social capital of Hungarian settlements to the risk of corruption in its local government, using large-scale social network data, finding that settlements with high bonding social capital tend to award contracts with higher corruption risk, while settlements with high bridging social capital tend to award lower corruption risk contracts. Berlusconi *et al*.^23^ tested link prediction techniques on the identification of missing links among an Italian mafia group and Ribeiro *et al*.^24^ made use of the same techniques on politicians cited on corruption scandals in Brazil.

In this work, we propose a network-based approach for analyzing bills-voting data in the form of representatives’ temporal networks to capture the topological structural changes along time and reveal how these changes may be reflected in (or *by*) some of the main political events happened during the same period in Brazil. Our analysis starts by converting each bill-voting session into a static network, in which each node represents a congressman and each edge represents the accumulated similarity of a pair of congressmen based on their historical votes on those bills. Afterwards, these static networks are converted to temporal networks by considering all of them as being an evolving network. We apply this technique to official data from the Brazilian House of Representatives, comprising the votes of 2,455 congressmen in a total of 3,407 bills-voting sessions from 1991 until 2019, hence covering a range of almost 30 years of legislative works. The obtained results are able to capture the main political transitions happened during the period in terms of the relative positions occupied by each political party in the network. We also find out that, surprisingly, the proposed technique is capable of identifying convicted representatives in the network with high precision and most of them are for corruption charges. This method can be used to predict cases of corruption or other financial crimes. Such a feature comes out unexpectedly since the networks’ edges are generated only based on the representatives’ legal public activities (bills-voting history), without any financial or other relative information of any sort.

In summary, this work makes use of specific dynamical measures for analyzing the Brazilian legislators’ networks. Moreover, it shows how the network-based framework can be applied to identify future cases of corruption or other financial crimes among congressmen with high accuracy, just based on the bills-voting data. Therefore, we believe this work makes an important advance in the large scale public data study using complex networks.

## Methods and Data

### Database used

The data are collected from the official website of the Brazilian House of Representatives^25^ within their transparency section. These datasets comprise the outcome of 3,407 voting sessions of legislative bills deliberated in the House of Representatives, from May 22, 1991 until Feb 14, 2019. We made a thorough data cleansing process in this database in order to detect and fix possible mistakes, such as duplicated names or votes and also typographical errors. Each voting session contains the following attributes: the bill to be voted, the voting date, and for each representative who attended the session: IDE (a unique number for each of them), Name, Party and Vote. The voting data are similar to roll call votes, except that here there are four different types of votes: (1) *Yes*, if the representative approves the bill; (2) *No*, if the representative disapproves the bill; (3) *Abstention*, if the representative deliberately chooses to not take part in the voting; and (4) *Obstruction*, similar to abstention, with the difference that abstention counts for *quorum* effects, i.e., the minimum number of voting members who must be present, while obstruction does not count for it.

After extracting and cleaning the data from the 3,407 voting sessions, we end up with a total number of 2,455 representatives and 1,656,547 votes. For analyzing these data, we opt for making use of a network-based technique, specially developed for this purpose. Firstly, we convert each voting session into a separated static network. Afterwards, we select some of these static networks to generate temporal networks and then perform some analyses in order to examine how their *topology* — in terms of network temporal measures — evolve along time.

As for the conviction classification task, also tested in this study, we add an additional attribute, for all representatives, which indicates whether he or she is currently convicted or have been arrested for corruption or other financial crimes, such as money laundering, peculation, embezzlement or misappropriation of public funds, improbity and crime against the Public Administration. This information has been confirmed from Brazilian judiciary official sources, such as the Federal Supreme Court (Supremo Tribunal Federal)^26^. At the end of this research, we were able to identify a total of 33 representatives in our database who currently have been either arrested or convicted for corruption (21 congressmen) or for other financial crimes (12 congressmen).

### Static network generation

A network can be defined as graph $G=({\mathscr{V}}, {\mathcal E} )$, where ${\mathscr{V}}$ is a set of nodes and ${\mathcal E}$ is a set of tuples representing the edges between each pair of nodes $(i,j):i,j\in {\mathscr{V}}$. The process of mapping each voting session in the database into a network is made according to their respective date attribute, sorted in ascending order, strictly. For the first voting, when $t=0$, its data items are initially converted to a square votes matrix *M*^*t*^ of size *dXd*, where *d* is the total number of representatives who participated in the session. Each element ${M}_{ij}^{t}$ is a binary value: it assumes assuming 1 if the vote of representative *i* is equal to the vote of representative *j*; otherwise, it assumes −1. These values are accumulated in a separated weight matrix *W*^*n*^, in which each element ${W}_{ij}^{n}$ is equal to the sum of values of ${M}_{ij}^{t}$ in all votes matrices *M*^*t*^ until voting session *n*. Hence, each item ${W}_{ij}^{n}$ of this matrix represents the accumulated weight between representatives *i* and *j*. The time steps *t* are measured in terms of voting sessions. Mathematically, the current value of each weight ${W}_{ij}^{n}$ is given by:

1 $${W}_{ij}^{n}=\mathop{\sum }\limits_{t=0}^{n}\,{M}_{ij}^{t}.$$

Hence, from Eq. (1), the values in each row ${W}_{i}^{n}$ may range from −*n*, in the case that the representative *i* always voted differently from representative *j*, until *n*, which is the case when *i* and *j* always voted alike. The former case implies that, up to the current instant, representatives *i* and *j* have complete opposite political views, while, in the latter case, *i* and *j* are very aligned up to now, politically speaking. Another possibility here, in this technique, would be binning the votes similarities per predetermined periods of time, such as per presidential term or per year. After some preliminary processing of the database, we have noted that it takes varying bills-voting time to emerge a clear topological pattern in the networks, therefore, it is suitable to take all historical votes into consideration for generating the weight matrix *W*^*n*^.

After generating the matrices *M*^*t*^ and *W*^*n*^, the next step is to generate a network *G*^*t*^, for each voting session *t*, such that each representative becomes a node in *G*^*t*^. The edges in *G*^*t*^ are created according to the following rule:

2 $${G}_{ij}^{t}=\{\begin{array}{ll}{W}_{ij}^{n}, & {\rm{if}}\,{W}_{ij}^{n}=\mathop{{\rm{\max }}}\limits_{\forall x\in {W}_{i}^{n}}\,x\\ 0, & {\rm{otherwise}}.\end{array}$$

As a result of Eq. (2), the great majority of the vertices in *G*^*t*^ will have only one outbound edge, connecting it to the most politically aligned vertex. Vertices with more than one outbound edge may only occur when the function ${{\rm{\max }}}_{\forall x\in {W}_{i}^{n}}\,x$ returns more than one value. The most connected vertices in the network (*hubs*) will be the ones with the highest number of inbound edges.

The pipeline of our technique can be summarized in the following steps:

1. build votes matrix *M*^*t*^ from data of voting session *t*;
2. update weight matrix *W*^*n*^, also inserting new representatives in it, if any;
3. build network *G*^*t*^, whose values come from the weight matrix *W*^*n*^; and
4. repeat the procedure for next voting session *t* + 1, until the last one in the dataset.

As a consequence of this process, the networks *G*^*t*^ evolve in time, as its edges are determined by the accumulated weights between pairs of representatives from matrix *W*^*n*^ and are updated at each step *t*. The vertices, representing the representatives, may also be replaced by new ones along the process, as new representatives appear in the voting session lists, such that the nodes, in this case, can be seem as the “chairs” in the Parliament. When a new congressman is inserted into the network (because he/she has been elected or for any other reason), he/she does not inherits any voting information from the congressman who previously occupied the chair of the House (or node in the network). In this case, the model adds a new row and a new column in matrix *W*^*n*^ to store the vote similarities between the node of the new congressman and all other nodes in *W*^*n*^. It is also worth noting that the attribute “party” is not taken into account by the model to generate the network’s edges. We proceed this way because, in this study, our aim is to capture the political affinities among representatives beyond their party affiliations, i.e., only taking into account their votes on legislative bills for network generation. This makes sense whereas, in the case of Brazil, there are currently as much as 35 different political parties, and this excessive number of parties ultimately makes the ideological differences among them to diminish substantially.

### Temporal network generation

**Figure 1:**
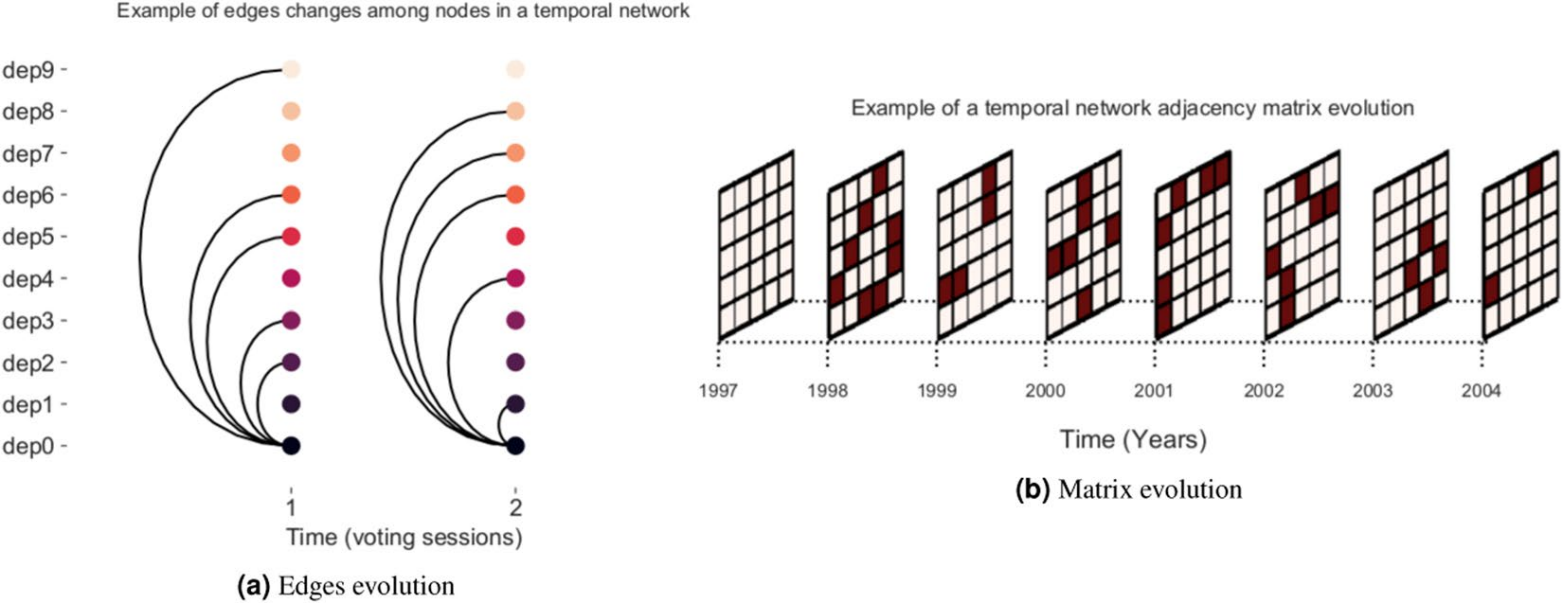
(**a**) Illustration showing how the temporal network edges, or *graphlets*, evolve in time, here measured in terms of bills-voting sessions. When time slice $t=1$, representative 0 is connected to representatives 2, 3, 5, 6 and 9. In the next time slice $t=2$, it loses the connections with representatives 2, 3, 5 and 9 and receives edges from representatives 1, 4, 7 and 8. (**b**) Example demonstrating the adjacency matrix evolution in a temporal network, whose dimension ${\mathscr{D}}$ is measured in units representing years. The network edges are generated according to this matrix.

After running our algorithm for all bills in the database, we end up to a total of 3,407 networks, each one with around 500 nodes and representing a different bill-voting session during the last 28 years. Thus, we can also say that all these networks, in fact, represent different moments of the Brazilian congressmen network. At this point, we already have shown how to generate these networks in a static form, each *G*^*t*^ representing a moment at time *t*. For the sake of converting these networks into a single temporal network *G*, we need then to insert a new dimension ${\mathscr{D}}$ in the static network definition, such that it becomes $G=({\mathscr{V}}, {\mathcal E} ,{\mathscr{D}})$, where ${\mathscr{D}}$ stands for the network temporal slices or, in our case, the voting sessions. To achieve this, we generate a matrix for representing each edge ${\mathcal E}$ in the static networks slices in the form of a triplet $(i,j,t):i,j\in {\mathscr{V}},t\in {\mathscr{D}}$. These triplets are also known as dynamic *graphlets*^27^ and an illustration of their dynamics is showed by Fig. 1a. The final result of this conversion process is a *multilayer network*, in which each layer represents a static temporal slice of a single main graph (Fig. 1b). In this case, since the dimension ${\mathscr{D}}$ is a set of indices ordered by time, we can therefore also call this graph a *temporal network*^28^, and perform analyses on it by extracting some specific measures.

Extracting temporal measures from a network with over 3,000 time slices, each one having around 500 nodes, is a time-consuming process. Therefore, in this work, we decide to make use of only one time slice per year for generating the temporal network. The selected bills-voting sessions, as well as the current presidency at each period and his/her corresponding political party, are described in Table 1. It is worth noting that the bills-voting sessions sampling (with one session in each year being selected as a temporal network slice) has little effect on the overall results, since our network formation technique certifies that the weight of each edge, stored in the weight matrix *W*^*n*^, already carries in itself the information regarding all previously voted legislative bills until present.

**Table 1:** Voting sessions used for generating the temporal network slices, yearly.

Year	Bill voted	Session date	Presidency
1991	PL 638/1991	1991-08-28	Collor (PRN)
1992	PL 2747/1992	1992-04-29	
1993	PL 1258/1988	1993-04-01	Itamar (PRN)
1994	PDC 413/1994	1994-04-20	
1995	PL 233/1995	1995-04-04	FHC I (PSDB)
1996	PL 824/1991	1996-04-10	
1997	PEC 173/1995	1997-04-09	
1998	PEC 33/1995	1998-04-29	
1999	PL 1/1995	1999-05-12	FHC II (PSDB)
2000	PEC 96/1992	2000-04-05	
2001	PLP 23/1999	2001-04-03	
2002	MPV 14/2001	2002-04-10	
2003	MPV 86/2002	2003-04-01	Lula I (PT)
2004	PEC 101/2003	2004-05-19	
2005	MPV 242/2005	2005-06-07	
2006	MPV 269/2005	2006-04-04	
2007	MPV 339/2006	2007-04-10	Lula II (PT)
2008	MPV 415/2008	2008-04-23	
2009	MPV 452/2008	2009-04-14	
2010	MPV 475/2009	2010-05-04	
2011	REQ. 343/2011	2011-04-06	Dilma I (PT)
2012	PEC 153/2003	2012-04-10	
2013	PEC 544/2002	2013-04-03	
2014	PLP 221/2012	2014-05-07	
2015	MPV 660/2014	2015-04-07	Dilma II (PT)
2016	REQ. 4250/2016	2016-04-04	
2017	PL 5587/2016	2017-04-04	Temer (MDB)
2018	PL 3734/2012	2018-04-11	

Besides generating one main temporal network, which includes all 28 time slices in Table 1, we also generate one temporal network per presidential term, for the sake of comparison purpose. The measures extracted from the resulting temporal networks are listed below.

- *Temporal degree centrality* (*D*^*T*^): the number of overall connections in time per node.
- *Temporal participation coefficient* (*P*^*T*^): a measure of diversity of connections across communities for individual nodes^29^. The communities are detected by using the Louvain method^30^.

We also calculate a “proportional” version of each temporal measure *M*^*T*^, grouped by the political party *p* of each node *i*, defined as:

3 $${M}_{p}^{T}=\frac{{\sum }_{i}\,{M}_{{i}_{p}=p}^{T}}{{\sum }_{i}\,{M}_{i}^{T}},$$

where *p* is a political party and *i*_*p*_ returns the party of node *i*. These proportional versions of the measures are used for comparison among parties.

### Conviction prediction

Now, let us proceed to describe how we assess whether a representative is more likely to be convicted or arrested in the future by analyzing the bills-voting agreements among congressmen. Two different methods have been tested for accomplishing this task: the first one is based on the matrix *W*^*n*^ values, while the second one is based on the network link prediction model. Following, we describe the two methods with more details.

#### Conviction prediction based on the weight matrix

After finishing the processing of all voting sessions, we end up with the network resulted from the final weight matrix *W*^*n*^. This network has 2,455 nodes, representing all congressmen who voted in at least one legislative bill from 1991 until 2019, along with their pairwise bills-voting similarities. While browsing this main network, we note that the highest weighted neighbors of a node labeled as convicted are more likely convicted ones as well, apparently forming some sort of “corruption neighborhoods” in the network. Hence, we decide to investigate this aspect further by running a very simple algorithm, which basically takes the *n* highest weighted neighbors of a convicted representative, according to the weights stored in *W*^*n*^, and labels all of them also as convicted ones. Thus, we have the “convicted” label *c* of a node *i* defined as follows:

4 $${i}^{c}=\{\begin{array}{ll}{\rm{True}}, & {\rm{if}}\,{j}^{c}={\rm{True}},\forall j\in k{{\rm{NN}}}_{i}\\ {\rm{False}}, & {\rm{otherwise}},\end{array}$$

where *k*NN_*i*_ returns the *n* neighbors with the highest weights associated to node *i*. We assess the efficiency of this model by measuring its prediction accuracy for different values of *n*. The rationale behind this model is that arrested or convicted representatives, for some reason, tend to vote similarly on legislative bills.

#### Conviction prediction based on link prediction

Given that the simple model described above does not consider the network topological structure for prediction purposes (only considers the weight matrix *W*^*n*^), we thus also test another method for accomplishing this task, which makes use of models for predicting missing links of the networks. The method’s pipeline is described below:

1. generate subgraph from an *undirected* version of the network from matrix *W*^*n*^, containing only arrested or convicted representatives and their neighbors;
2. remove all existing links between convicted labeled nodes from this network (subgraph);
3. apply link prediction model to the network; and
4. take the top *n* link predictions whose source is a convicted labeled node and classify their target nodes also as convicted ones.

One of the models tested for this task is Rooted PageRank^31^, which is based on an algorithm developed for ranking the importance of website pages^32^. It defines the *score*(*x*, *y*) as the expected number of steps required for a *random walk* on the network starting from node *x*, moving iteratively with a probability *α* to return to *x* (or “reset”) and a probability 1 − *α* to move forward to a random neighbor until it reaches the node *y*. The lower the *score* for each pair of nodes *x* and *y* is, the higher the pair is ranked among the model’s predicted links. Besides Rooted PageRank, other 5 link prediction models are also applied to this task: Pearson^33^, Cosine^34^, NMeasure^35^, MinOverlap^36^ and Random (for comparison purposes). By making use of a link prediction model, we are now taking into account the congressmen network topological structure for conviction prediction purposes.

## Results and Discussion

### Political scenario through the analysis of the representatives’ networks

**Figure 2:**
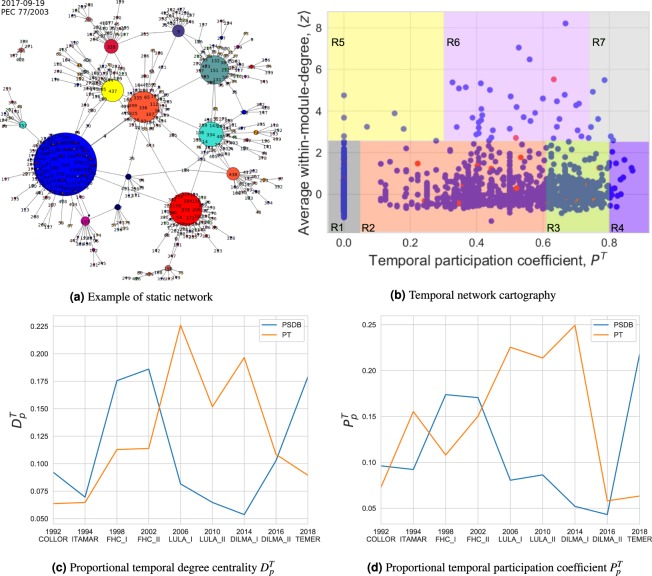
(**a**) Example of a static network generated by our algorithm for the voting session occurred on 2017-09-19 of legislative bill PEC 77/2003. Each node represents one of the 513 congressmen who voted this bill and each color represents a different political party. (**b**) Node roles based on the network cartography framework^29^, with the adaptation that, here, we use the temporal version of the participation coefficient (${P}^{T}$) with averaged within-module-degree, z-scores, from each temporal network slice $t$. Each point represents a congressman and the *red* color denotes convicted ones. (**c**) Proportional temporal degree centrality ${D}_{p}^{T}$ and (**d**) proportional temporal participation coefficient ${P}_{p}^{T}$ measures evolution, calculated for all representatives and grouped by political party $p$, for each presidential term. The evolution of both measures coincide precisely with the respective alternation of the ruling parties PSDB (FHC) and PT (Lula and Dilma).

As mentioned earlier, our initial task involves the generation of over 3,000 static networks in total, then, a comprehensive temporal network is built. We start this subsection by presenting an example of one of these static networks shown by Fig. 2a, built from the voting session of legislative bill PEC 77/2003, occurred on September 19, 2017. The outbound edges connect each node to the one with the highest accumulated weight associated with it. One feature that called our attention in most of these networks is that, even though the *party* attribute, represented by the color of the nodes in the figure, has not been taken into account explicitly by the algorithm, we still can note the formation of neighborhoods based on parties in the networks, centered at *hubs*. This feature confirms that representatives from the same party tend to vote alike in legislative bills, thus, the formation of party clusters occurs. If a node is connected to a neighborhood different from its own party’s, then the congressman represented by this node has been voting more similarly to the representatives of other parties. As expected, still in Fig. 2a, the colors of the biggest hubs in the network coincide to those from the parties with most members in the House of Representatives at that time. The colors in *blue*, *red*, *cadet-blue* and *orange* represent the parties PSDB, PT, PP and MDB, respectively, which were main parties in the Brazilian congress in September 2017. The hubs, within this context, represent the congressmen who voted according to each “local majority” in the network, i.e., the majority within a local neighborhood.

Alternation of power is an important and expected condition of democratic systems. Within this context, we analyze the temporal networks segmented by each presidency, with the aim of measuring the evolutionary strength of the two main political parties (PSDB and PT) in Brazil during the considered period, in terms of the positions they occupy in the network, and examine how these changes are related to the main political events happened in the same period. We initially extract two centrality measures from each network: temporal degree centrality *D*^*T*^ and temporal participation coefficient *P*^*T*^, which give us centrality scores for each node. Afterwards, we calculate the ratio of each of those measures for the parties PSDB and PT, according to Eq. (3), in each presidential term. For the cases when a representative switched parties during the period, we then consider the party to which he belonged at the time of each voting session, i.e., each time slice. The results of this process are shown by Fig. 2c,d. Observe that the ruling political party presents higher values for both centrality scores measured in the congressmen temporal network and such a feature strictly follows the respective alternation of power between PSDB (FHC governments, from 1995 until 2001) and PT (Lula and Dilma governments, from 2002 until 2016). It is also worth noting that, in these figures, there is a sudden drop in both measures for the PT party in the second term of Dilma (2015–2016), which coincides with the turbulent political scenario in Brazil at that time, when many demonstrations were held against Dilma — specially after her predecessor Lula was charged by federal prosecutors with corruption accusations against him and his party — and end up in her impeachment, in 2016. A similar behavior can be also observed for the PTC party (former PRN) in 1992 (the impeachment of former president Collor occurred at that time) in a much smaller scale since this is a minor political party in Brazil. This event is not included in these figures for the sake of visibility.

Following, we generate what is known as the *network cartography*^29^ for the temporal network which includes all 28 time slices (from 1991 until 2018, yearly). This framework helps us to better understand the network topological structure by grouping the nodes into some “universal roles”, according to their level of connectivity inside the network. It depends on two measures: the *within-module degree z*_*i*_, which shows how “well-connected” a node *i* is to other nodes within its module, and the *participation coefficient P*_*i*_, which shows how “well-distributed” the links of node *i* are among different modules. For accomplishing this task, we make a slight adaptation from the original technique. For static networks, the within-module-degree returns a single value *z*_*i*_ for each node *i*. As for temporal networks, instead, it returns a 2-D array in the form of *z*_*it*_ with one value of *z*_*i*_ for each time slice *t*. Therefore we opt here for averaging these values, such that ${z}_{i}=\overline{{z}_{it}}$ in order to generate the network cartography. We also make use of the temporal participation coefficient ${P}_{i}^{T}$, instead of its static version *P*_*i*_. The output can be seen in Fig. 2b. Each point in this plot represents a congressman and the *red* color denotes those nodes labeled as convicted ones. The distribution of their network roles is summarized in Table 2, grouped by arrested or convicted and the others (those who have not been arrested or officially convicted). It shows that around 98% of them are non-hubs (roles R1 to R4) and only about 2% of them are module hubs (roles R5 to R7), indicating that convicted representatives tend to have a slightly higher incidence of *connector hubs* (R6), which are hubs with links to most of the other modules.

**Table 2:** Network cartography: node roles distribution (%).

Role	Convicted	Others	Description
R1–ultra-peripheral	62.0	63.1	nodes with all their links within their module
R2–peripheral	26.0	25.4	nodes with most links within their module
R3–non-hub connector	9.8	9.7	nodes with many links to other modules
R4–non-hub kinless	—	0.5	nodes with links homogeneously distributed among all modules
R5–provincial hubs	—	0.2	hubs with the vast majority of links within their module
R6–connector hubs	2.2	1.0	hubs with many links to most of the other modules
R7–kinless hubs	—	0.1	hubs with links homogeneously distributed among all modules

### Prediction of conviction among representatives

The incidence of corruption impacts the society negatively in many ways, such as holding back businesses, wasting public spending and undermining the democratic system. Predicting the incidence of corruption and other related financial crimes, specially at the individual level, is a challenging task. Nowadays, a prediction system with an average accuracy around 0.2 is already considered useful by public investigators all over the world^24^. Here, we make use of a network-based approach to identify hidden connections among convicted congressmen linked to bribing schemes or other financial crimes in Brazil. Two methods are tested for detecting future convictions among representatives. The first method is based on the nearest neighbor of convicted congressmen using the weight matrix *W*^*n*^ and the second one is based on link prediction. The former achieves prediction accuracy about 0.24, while the latter achieves accuracy beyond 0.5, even up to 0.9. Consequently, the accuracy obtained by the link prediction model can be considered quite satisfactory. The reason why the prediction accuracy by the two methods are so different is simple: In the first method, a prediction to a congressman is made by considering only his/her labeled nearest neighbor, i.e., a prediction is conditioned on only one node of the network. On the other hand, in the second method, a prediction is made by link prediction methods, which considers local or global network structure conditioned on more than one nodes, i.e., a finer filtering is performed.

#### Results based on the weight matrix

**Figure 3:**
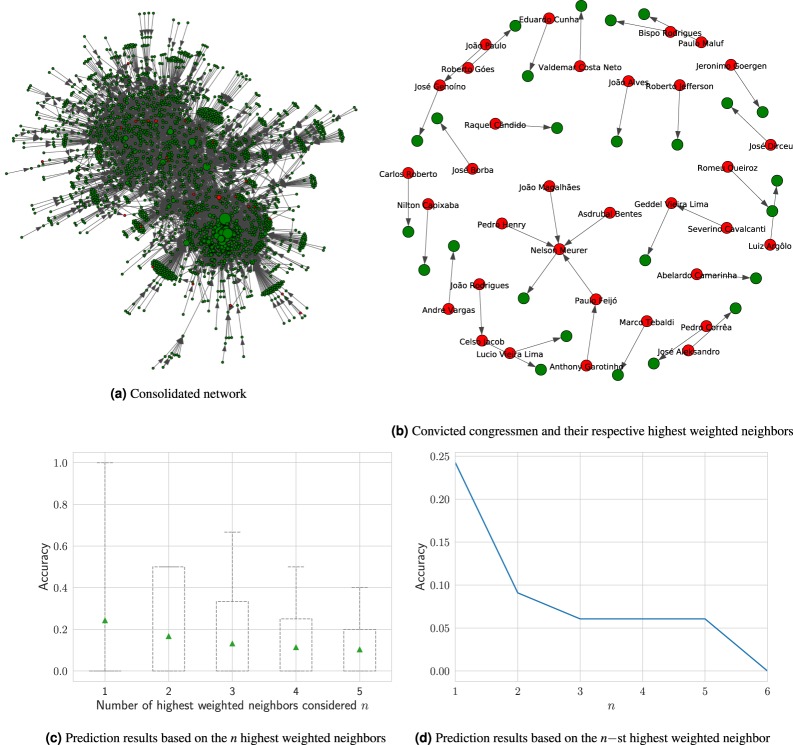
(**a**) Representation of the network resulted from the final matrix *W*^*n*^, with all 2,455 congressmen in the database, disregarding the time factor. Each node is connected to its highest weighted neighbor, in terms of votes similarity on legislative bills. The red color denotes convicted representatives (33 in total). (**b**) A subgraph of the consolidated network, showed in (**a**), displaying only the 33 already arrested or convicted representatives (in red) and their respective highest weighted neighbors. We opted for not displaying the names of representatives who currently have not been arrested or officially convicted in this graph (in green). (**c**) Predictions based on the $n$ highest weighted neighbors, in terms of votes similarity, resulted in an average accuracy of 0.243 when $n=1$. (**d**) Tests made by considering the *n*–st highest weighted neighbor of a convicted node show that, as we increase the value of $n$, the lower is the average accuracy.

While browsing the nodes of the network resulting from the final weight matrix *W*^*n*^ (Fig. 3a) — the one formed by all representatives, regardless the time factor — the first speculation in mind may be that the highest weighted neighbors of a convicted corrupt representative are possibly convicted ones as well. Therefore, we investigate whether the nodes of arrested or convicted representatives tend to stay close to each other in this network, and thus forming some sort of “corruption neighborhoods”, so to speak. For this purpose, we build *n* separated networks composed only by nodes labeled as convicted ones, along with their respective *n* highest weighted neighbors according to the final weight matrix (these neighbors can be labeled as convicted or not). Afterwards, we run a simple algorithm, as specified in Eq. (4), which classifies all *n* neighbors of an already convicted labeled node as being convicted ones as well (whether in the present or in the future). In Fig. 3b, it is possible to see the network resulted from *n* = 1, i.e., with the 33 convicted representatives along with the highest weighted neighbor of each of them. It indicates that there is, indeed, the formation of some sort of “corruption structures” in the network. Note that Fig. 3b is actually a subgraph of Fig. 3a, which has 2.455 nodes and only 33 of them labeled as convicted. So the odds of a convicted node having a neighbor who is also convicted would be very low, if it is not for the incidence of the corruption neighborhoods. The emergence of this feature is something surprising to us, considering that none of the input attributes in our data are related to the congressmen’s financial income or expenditures and that the edges are generated solely based on their bills-voting history. The conviction prediction results for *n* in $[1,5]$ are shown by Fig. 3c. From this figure, we see that the optimal value of *n* is 1, with an average accuracy of 0.24.

In order to confirm whether there is indeed a correlation between bills-voting similarity and convictions for corruption and other financial crimes among representatives, we run another test by using the same rationale explained above with the difference that, here, instead of selecting the highest weighted neighbor of each convicted node for prediction purposes, we took its *n*–st highest weighted neighbor determined by its outgoing edges, therefore decreasing the votes similarity between the original convicted node and its neighbor, as *n* increases. The obtained results, in Fig. 3d, show that, in this case, the higher the value of *n*, the smaller is the accuracy achieved by the algorithm, which contributes to confirming our initial suspicion that convicted representatives indeed tend to vote alike in legislative bills.

The prediction accuracy achieved by our first prediction model is about 0.24 and it is very close to the accuracy achieved by Ribeiro *et al*.^24^, which is around 0.26, when predicting missing links among politicians cited on corruption scandals in Brazil. Following, we show how the prediction rate can be considerably improved when we take into account the overall network topological structure for making the predictions.

#### Results based on a link prediction model

The last step in our analyses involves the application of link prediction techniques for the sake of predicting new conviction cases among the representatives. For accomplishing this task, we apply a total of 5 link prediction models plus a Random method (for comparison purposes) in the congressmen network. The method based on link prediction differ from the simple one presented in the previous sub-section because the former makes a prediction considering the network’s topological structure (excluding, of course, the random technique from this list), while the latter just takes into account certain neighbors. As in the previous test, the models are also applied to a subgraph of the network resulted from the final weight matrix *W*^*n*^ formed by convicted representatives and their respective neighbors, with the difference that, at this time, neighbors from both incoming and outgoing edges are considered, and also that the network is previously converted to an *undirected* one. The final subgraph contains 211 nodes (33 of them being convicted) and 1,374 edges. As a preprocessing, we remove all existing links between two nodes labeled as convicted from the network (5 in total). After running the link prediction models, we took the top *n* predicted links with convicted nodes as sources and label their target nodes as being convicted ones as well. All the tests are performed using the tool introduced by Guns^37^ with default parameters values for all models.

**Figure 4:**
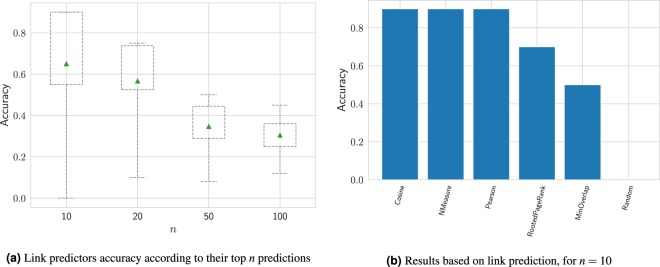
(**a**) Performances achieved by 6 link prediction models on the task of predicting conviction cases among representatives by considering the top $n$ predicted links whose source node is a convicted one, indicating that the highest scores are achieved when $n=10$, with an average accuracy of 0.65. (**b**) Performances achieved by each model, when considering their top 10 predictions, showing Cosine, NMeasure and Pearson with the highest score, with an impressive accuracy of 0.9.

The obtained results of all 6 link prediction models under consideration are shown by Fig. 4a,b. Figure 4a shows how the value of *n*, in this case, may affect the overall results, where *n* = 10 is the most indicated among the tested values, with an average accuracy of 0.65 (around 6 correct ones out of every 10 predictions, then). Figure 4b brings the accuracy achieved by each model, with Cosine, NMeasure and Pearson showing an impressive performance with an accuracy of 0.9, followed by Rooted PageRank and MinOverlap, with an accuracy of 0.7 and 0.5, respectively. It is worth noting that the Random predictor scored 0 in this task, which contributes to highlighting the effectiveness of applying the graph-structure-based predictors.

**Figure 5:**
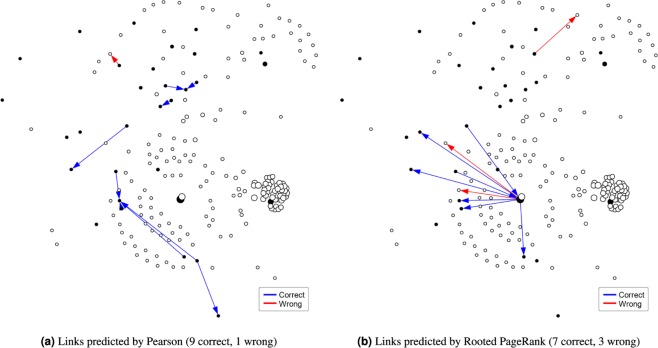
Comparison of two link prediction outputs for the network formed by convicted representatives and their neighbors: top 10 links having a convicted node as source predicted by (**a**) pearson and (**b**) rooted PageRank models. Black nodes indicate convicted ones. A link prediction is considered correct if its target node is also labeled as convicted. Remembering that the models do not take the node labels into account for prediction purposes. All other links are removed from the network only for the sake of visibility.

Comparing between the first prediction model with the average accuracy of 0.24 and the link prediction models with accuracy beyond 0.65, we perceive how the performance of a model can be improved whereas one considers the topological structure of the input dataset for classification purposes. This feature becomes more evident given the good results achieved by the first 5 link prediction models shown in Fig. 4b. The performance of link predictors, overall, may vary significantly, with some methods being more suitable than others according to the input dataset^31^. In our case, given the technique used to build the congressmen network, two features have emerged from it: (1) the more politically aligned two representatives are (in terms of their votes on legislative bills), the nearer they are in the network (in terms of number of links); and (2) only long term representatives are able to become hubs in the network, since a higher number of votes on bills is needed for that. In Fig. 5, we show a comparison of the top 10 link predictions from the Pearson and Rooted PageRank models for the network. This figure may help us to better understand why some link-prediction-based methods performed different than others in the task of predicting new convicted nodes. Methods such as Pearson, Cosine and NMeasure have in common the fact of being *local predictors*, i.e., solely based on the neighborhoods of the two nodes considered. Hence, they presented very similar results, also achieving the best accuracy when compared to other methods. This may be related to the feature where convicted nodes tend to stay close to each other in the network, as we saw earlier. As for the Rooted PageRank, which achieved the second best accuracy of 0.7, it is a *global predictor*, such that even if two nodes do not share any common neighbors, they still may be related and form a link in a later stadium. One may observe that all 7 correct links predicted by Rooted PageRank have the largest network hub (the one in black, in the center) either as its source or as its target and, in this case, it also happens that the largest hub in the network is a convicted one himself. This feature favors models based on random walks, such as Rooted PageRank, since many of the other convicted nodes are close to this hub.

## Discussions

Fighting and preventing corruption and other financial crimes are challenging tasks, because criminals constantly develop increasingly advanced mechanisms to cover their infractions. In this study, we present a technique to reveal the hidden relationships between bills-voting behavior and condemnations for corruption and other financial crimes among politicians. We also show how this information can be used to detect those individuals which are more likely to be convicted in the future. To our knowledge, this work is one of the first endeavours to accomplish such task through a network-based methodology. An interesting feature of this work is that the high conviction-prediction accuracy can be obtained using bills-voting data, which implies that it is possible to reveal politicians’ illegal behavior through just their legal public activities. Such kind of systems, once is developed, is certainly quite useful to many countries, specially to the countries like Brazil, which seriously suffer from corruption.

Our work is inspired by Ribeiro *et al*.^24^, which predicts missing links among politicians cited on corruption scandals in Brazil. Both works (the one of Ribeiro *et al*.^24^ and our work) deal with a similar problem — the incidence of corruption among individuals by using network-based techniques. However, there is a fundamental difference between the two works: The former is based on a dataset composed of 404 politicians cited on at least one corruption scandal and aims to predict citations on future scandals, while our study is based on a dataset comprising the voting history of 2,455 representatives on legislative bills and only considers those already arrested or found officially guilty for prediction purposes. Therefore, the dataset used in this work is not only a larger one, but also is always available and easy to access. The use of regular public data, as the dataset we use here, presents big facility to develop politician monitoring system in the future. Besides of this, the prediction accuracy achieved by our prediction model, about 0.9, is much higher than that obtained in Ribeiro *et al*., which is around 0.26. We hence believe that the accuracy rate achieved in this work is quite satisfactory. Another related work, of Berlusconi *et al*.^23^, tested link prediction techniques based on a similarity score on the identification of missing links among an Italian mafia group, obtaining a link reliability of up to around 0.9 for predictions made based on common neighbors. However, the prediction accuracy has been counted, in some cases, by considering informal relationships among the members of the mafia, for example, the existence of a phone call between the two members (two nodes), which presents certain level of subjectivity. On the other hand, in our work, the corruption prediction accuracy is calculated using official judiciary sources, such that we are certain whether a congressman is convicted or arrested. It means that we are sure with the prediction accuracy of our model.

As future works, we plan to extract other measures from the temporal network, such as the temporal betweenness centrality, the temporal closeness centrality and bursting measure, in order to better understand its topological structure. Other network building methods will also be developed to include more relevant information of the congressmen, such as the federal state of each of them represents, original profession, sex, age, kinship among them, and so on. For the conviction prediction task, one can, for example, filter the representatives’ historical votes by types of bills and then identify which kinds of bills are more likely to lead to corruption and other financial crimes. So we can alert people to pay more attention to those kinds of bills. Finally, we believe our work contributes to the development of big data platform to monitor politicians’ behavior.

## Data Availability

The datasets generated during and/or analyzed in the current study are available from the corresponding author on reasonable request.
